# 2,2-Difluoro Derivatives of Fucose Can Inhibit Cell Surface Fucosylation without Causing Slow Transfer to Acceptors

**DOI:** 10.1021/jacsau.4c00681

**Published:** 2024-09-23

**Authors:** Yanyan Liu, Igor R. Sweet, Geert-Jan Boons

**Affiliations:** † Chemical Biology and Drug Discovery, Utrecht Institute for Pharmaceutical Sciences, Utrecht University, 3584 CG Utrecht, The Netherlands; ‡ Complex Carbohydrate Research Center, University of Georgia, 315 Riverbend Road, Athens, Georgia 30602, United States; § Bijvoet Center for Biomolecular Research, Utrecht University, 3584 CG Utrecht, The Netherlands; ∥ Chemistry Department, The University of Georgia, Athens, Georgia 30602, United States

**Keywords:** glycans, synthesis, inhibitor, fucosylation, fucosyltransferase, prodrugs

## Abstract

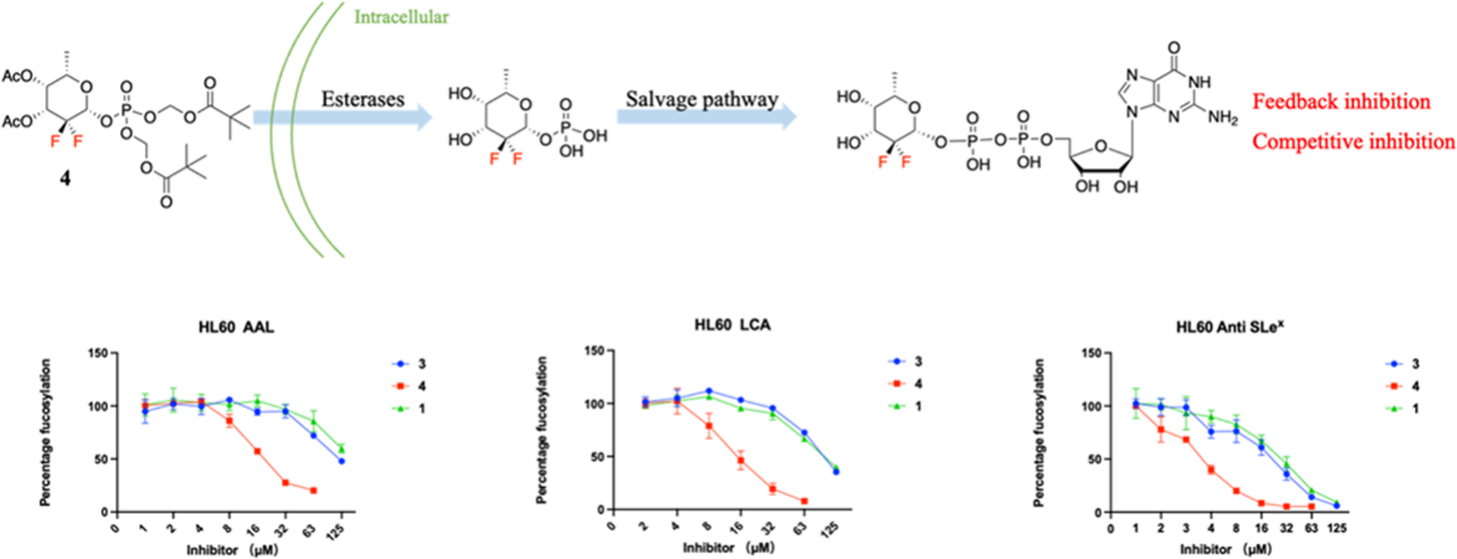

Fucosyl transferases (FUTs) are enzymes that transfer fucose (Fuc) from GDP-Fuc to acceptor substrates, resulting in fucosylated glycoconjugates that are involved in myriad physiological and disease processes. Previously, it has been shown that per-*O*-acetylated 2-F-Fuc can be taken up by cells and converted into GDP-2-F-Fuc, which is a competitive inhibitor of FUTs. Furthermore, it can act as a feedback inhibitor of *de novo* biosynthesis of GDP-Fuc resulting in reduced glycoconjugate fucosylation. However, GDP-2-F-Fuc and several other reported analogues are slow substrates, which can result in unintended incorporation of unnatural fucosides. Here, we describe the design, synthesis, and biological evaluation of GDP-2,2-di-F-Fuc and the corresponding prodrugs as an inhibitor of FUTs. This compound lacks the slow transfer activity observed for the monofluorinated counterpart. Furthermore, it was found that GDP-2-F-Fuc and GDP-2,2-di-F-Fuc have similar *K_i_* values for the various human fucosyl transferases, while the corresponding phosphate prodrugs exhibit substantial differences in inhibition of cell surface fucosylation. Quantitative sugar nucleotide analysis by Liquid chromatography–mass spectrometry (LC–MS) indicates that the 2,2-di-F-Fuc prodrug has substantially greater feedback inhibitory activity. It was also found that by controlling the concentration of the inhibitor, varying degrees of inhibition of the biosynthesis of different types of fucosylated *N-*glycan structures can be achieved. These findings open new avenues for the modulation of fucosylation of cell surface glycoconjugates.

## Introduction

**Figure 1:**
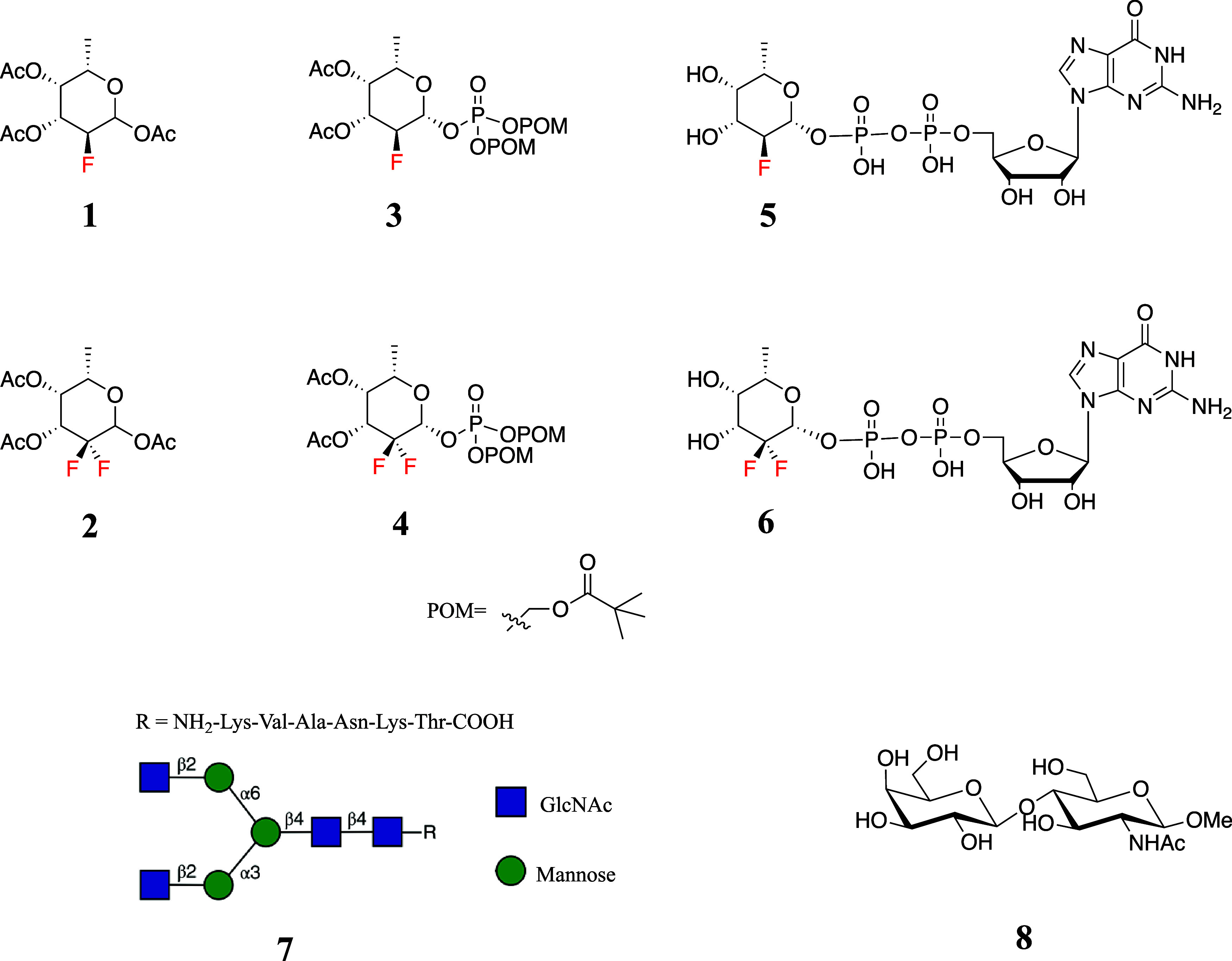
Chemical structures of inhibitors of FUTs and substrates for testing their inhibitory activity.

Fucosylation of glycoproteins and glycolipids is important for many biological processes such as cell signaling, cell adhesion, cell differentiation, immune response modulation, and pathogen recognition fucosyl transferases (FUTs) that catalyze the transfer of fucose (Fuc) from guanosine diphosphate β-l-fucose (GDP-Fuc) to an acceptor glycoprotein or glycolipid.^1−3^ FUT1 and FUT2 are α1,2-fucosyl transferases responsible for the synthesis of H-type blood group antigen and related structures, whereas FUT3, FUT4, FUT5, FUT6, FUT7, and FUT9 synthesize α1,3- and α1,4-fucosylated glycans, which are part of Lewis and blood group antigens.^1,4^ FUT8 is the only α1,6-fucosyltransferase responsible for the core fucosylation of *N*-glycans. Despite substantial interest, inhibition of FUTs for research or therapeutic purposes remains challenging.^5−8^ Two main approaches have been pursued for the development of inhibitors for the FUTs. The first approach involves high-throughput screening of compounds that structurally are not related to fucosyl transfer substrates,^9,10^ some of which exhibit *K_i_* values in the nanomolar range.^11^ Due to a lack of structural similarity to fucose, these compounds may exhibit off-target effects. A second direction focuses on substrate analogues as competitive inhibitors of GDP-Fuc.^5,7,8^ Notable examples include GDP-2-F-Fuc,^6^ GDP-6-F-Fuc,^6^ and GDP-carba-Fuc.^12,13^ However, the high negative charge of nucleotide sugar analogues hampers efficient penetration of cell membranes, limiting utility in living cells. Various approaches have been explored to overcome this limitation, which include the development of per-acetylated-Fuc derivatives and phosphate prodrugs.^14−18^ A key discovery was that per-*O*-acetylated 2-F-Fuc (1, Figure 1) can be taken up by cells and be converted into GDP-2-F-Fuc (**5**) by enzymes of the salvage pathway.^14^ The resulting compound is a competitive inhibitor of GDP-Fuc, thereby blocking the activity of FUTs. Furthermore, it is a feedback inhibitor of *de novo* synthesis of GDP-Fuc that in turn reduces glycan fucosylation.

The potency of GDP-2-F-Fuc is modest, often requiring high concentrations (∼200 μM) for a substantial reduction in cell surface fucosylation. Moreover, GDP-2-F-Fuc acts as a slow substrate for several FUTs,^6,14^ which may result in unintentional incorporation of the modified fucoside into glycan acceptors. Other fucose derivatives that can be taken up by cells and converted into GDP-Fuc analogues are also slowly transferred to glycan acceptors.^15,16,19^

We reasoned that GDP-2,2-di-F-Fuc (**6**, Figure 1) may be a competitive inhibitor of fucosyl transferases. It was expected that this compound would be resistant to slow transfer to glycan acceptors because the additional electron-withdrawing fluorine atom at C2 further destabilizes the partial positive charge that is developed in the transition state of transfer of Fuc of GDP-Fuc to an acceptor substrate.^20^ For intracellular applications, prodrugs **2** and **4** were prepared and evaluated. The incorporation of the bis(pivaloyloxymethyl) (POM) group was inspired by the successful application of several FDA-approved antiviral drugs such as adefovir dipivoxil.^21^ The POM carbonate can be removed intracellularly by esterases, resulting in an unstable carboxylate intermediate that undergoes chemical degradation to a phosphate monoester. The POM moiety has been successfully applied as a prodrug for 6-CF_3_–Fuc.^16^ 2-F-Fuc derivatives **1** and **3** were also synthesized for comparison purposes. Compounds **7** and **8** were prepared to function as acceptors of various human FUTs.

We found that GDP-2,2-di-F-Fuc (**6**) is a potent inhibitor of various human FUTs and can substantially reduce cellular GDP-Fuc concentrations by the inhibition of *de novo* biosynthesis. Peracetyl-2,2-di-F-Fuc (**2**) did not reduce cellular fucosylation while prodrug **4** exhibited potent inhibition of fucosylation of cell surface glycoproteins. Furthermore, it was found that this compound is much more potent compared to monofluorinated **1** and **3**. Importantly, glycosyl acceptor **7** could not be modified by GDP-2,2-di-F-Fuc (**6**) upon exposure to FUT8 for extended periods of time. Collectively, the results demonstrate that difluorination of fucose eliminates the risk of unintended incorporation of the fucose analogue into glycoproteins and gives compounds of higher inhibitory activity in cell-based studies.

## Results and Discussion

### Preparation of Fucosylation Inhibitors

**Scheme 1:**
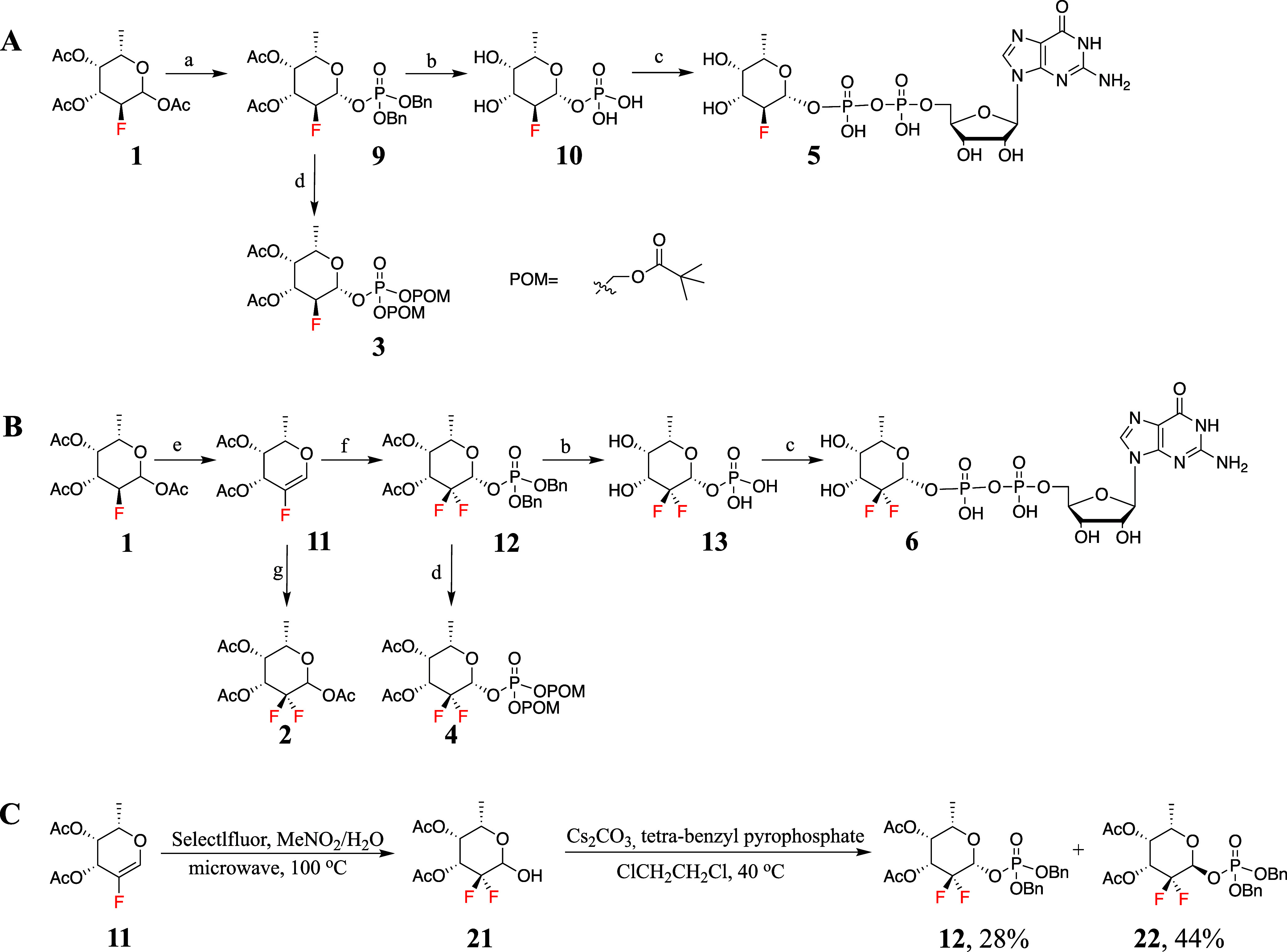
(A): Synthesis of Mono-F Derivatives (a) i. HBr/AcOH, DCM, ii. Ag_2_CO_3_, (BnO)_2_POOH, 3 Å MS, CH_3_CN, 70% (b) i. H_2_, Pd/C, MeOH, ii. MeOH, H_2_O, Et_3_N, 88% (c) GMP-Morpholidate, 1*H*-tetrazole, Pyridine, 18% (d) i. H_2_, Pd/C, MeOH, ii. Ag_2_CO_3_, POMCl, CH_3_CN, 60 °C, 66%. (B): Synthesis of Difluoro Derivatives (e) i. HBr/AcOH, ii. Et_3_N, CH_3_CN, 82 °C, 52% (f) i. Selectfluor, MeNO_2_/H_2_O, Microwave Heating, 100 °C, ii. Cs_2_CO_3_, Tetrabenzyl Pyrophosphate, ClCH_2_CH_2_Cl, 40 °C, 28% β-anomer (g) i. Selectfluor, MeNO_2_/H_2_O, Microwave Heating, 100 °C, ii. Ac_2_O, Pyridine, 85%. (C): Detailed Reaction Process for the Conversion of **11** into **12**, Which is Part of Panel (B)

First, we synthesized the series of 2-F-Fuc derivatives (Scheme 1A).^6,14,22^ Compound **1** was treated with hydrogen bromide in acetic acid resulting in the formation of fucosyl bromide which was reacted with dibenzyl phosphate in the presence of silver carbonate resulting in S_N_2 displacement of the bromide to afford **9** as only the β-anomer in a yield of 70% over 2 steps. Compound **9** was deprotected by a two-step procedure entailing hydrogenation over Pd/C and deacetylation by Et_3_N, resulting in the formation of **10** in a yield of 88%. Compound **10** was coupled with GMP-morpholidate in pyridine and catalyzed by 1*H*-tetrazole leading to the desired GDP-2-F-Fuc (**5**) in a yield of 18%. The low yield of this step is common for this type of condensation and proceeds slowly with substantial starting materials remaining even after a prolonged reaction time of 5–7 days which is followed by a challenging purification. 2-F-Fuc-prodrug **3** was synthesized from **9** by a two-step procedure involving removal of the benzyl esters of the phosphate by catalytic hydrogenation over Pd/C to give a monophosphate that was protected by POM by reaction with POMCl in acetonitrile at 60 °C (66% yield, two steps).

The synthesis of the 2,2-di-F-Fuc derivatives is outlined in Scheme 1B, which involved a vinyl fluoride as a key intermediate for the introduction of the second fluorine atom at C2.^23−25^ We explored several methodologies, such as constructing the fucose ring from a four-carbon difluoro derivatives.^26^ However, these approaches were ineffective, and we focused on an approach involving a vinyl fluoride intermediate. Thus, treatment of **1** with hydrogen bromide in acetic acid resulted in the formation of an intermediate anomeric bromide that was treated with triethylamine in acetonitrile to induce elimination and formation of vinyl fluoride **11** in a yield of 52%.^27^ Electrophilic fluorination of **11** with Selectfluor in a mixture of nitromethane and H_2_O, followed by acetylation of the resulting anomeric hydroxyl produced per-*O*-acetyl-2,2-di-F-Fuc (**2**) in a yield of 85%. To achieve this result, the reaction condition for this transformation was optimized. Heating the reaction mixture in an oil bath ranging from 40 to 80 °C for several hours to overnight resulted in product decomposition while the starting material was not fully consumed, thus making it difficult to determine the optimal reaction time. At various time points (4, 8, and 18 h), the compound was isolated, but in each case, the yield was less than 50%. The use of a microwave reactor instead of traditional heating reduced the reaction time to just 5–10 min, greatly enhancing the reaction efficiency and yield.

Next, attention was focused on the preparation of compound **12**, which is a key intermediate for the synthesis of target compounds **4** and **6** (Scheme 1B,C). The generation of the β-anomeric phosphate linkage of **12** proved challenging, and most of the examined reaction conditions gave only the α-anomer. This agrees with an earlier observation that methoxycyclohexanes favor an axial conformation when a CF_2_ group is placed at the C-2, C-4, and C-6 positions due to a pseudoanomeric effect.^28^ Anomeric acetate **2** was also isolated as the main α-anomer, and it appeared that during storage it further isomerizes to the α-anomer, further supporting the preference of the axial anomer. The difluoro modification at C2 also greatly increases the stability of the chemical bond at C1, making the installation of a leaving group at C1 challenging. Instead of generating an oxocarbenium ion intermediate for anomeric phosphorylation, 2,2-di-F-Fuc lactol **21** was employed as a nucleophile for attack on a phosphate anhydride in the presence of a base to form compound **12**. Thus, compound **11** was subjected to electrophilic fluorination using Selectfluor in a mixture of nitromethane and H_2_O which resulted in the formation of compound **21**, which is rather unstable and decomposed during purification and therefore was immediately employed in the next step (Scheme 1C). Various reaction conditions were explored to convert **21** into **12**, including different bases (such as DMAP and *n*-BuLi), phosphorylation reagents (such as P(OBn)_2_N(*i*Pr)_2_ and ClPO(OPh)_2_), and temperatures (such as room temperature, 0 and −78 °C), which gave the α-anomer with negligible β-anomer formation. Based on these results, we presumed that the α-anomer is the thermodynamic while the β-anomer is the kinetic product. Therefore, we expected that the use of a relatively weak base and a phosphorylation reagent of lower activity may enhance the β-anomeric selectivity.^27,29−31^ By utilizing cesium carbonate as the base and tetrabenzyl pyrophosphate as the phosphorylation reagent at 40 °C, a separable mixture of the α-anomer (*J*_H1-Fax_ = 6.5 Hz, 44%, **22**) and β-anomer was obtained (*J*_H1-Fax_ = 14.3 Hz, 28%, **12**). *F*_ax_ could be distinguished from *F*_eq_ by the large coupling constant with H-3 (*J*_H3-Fax_ = 20.7 Hz), and as a result, the anomeric configuration could be determined by comparing the coupling constant of H-1 with *F*_ax_. Higher reaction temperatures, such as 60 and 80 °C, resulted in lower yields of both the α-anomer (10%) and the β-anomer (10%), while at room temperature, almost only the α-anomer (43%) was produced.

Compound **12** was deprotected by a two-step process involving hydrogenation over Pd/C followed by deacetylation with Et_3_N yielding compound **13** in 88% yield. Subsequently, compound **13** was condensed with GMP-morpholidate in pyridine and catalyzed by 1*H*-tetrazole, resulting in the production of desired GDP-2,2-diF-Fuc (**6**). 2,2-diF-Fuc prodrug **4** was synthesized from **12** by removal of the benzyl protecting groups by hydrogenation over Pd/C, resulting in a monophosphate group intermediate that was acylated with POMCl resulting in **4** in a yield of 66% over 2 steps.

The preparation of glycosyl acceptors **7** and **8** is described in the Supporting Information (Figure S1 and Scheme S1).

We also investigated enzymatic approaches to synthesize compounds **5** and **6** (Scheme S2). Bacterial l-fucokinase/GDP-fucose pyrophosphorylase (FKP) is a bifunctional enzyme that catalyzes the conversion of fucose to Fuc-1-P and subsequently to GDP-Fuc. FKP exhibits promiscuity toward l-fucose derivatives including 2-F-Fuc.^14,32^ Compound **5** could easily be synthesized by treatment of 2-F-Fuc with ATP and GTP in the presence of FKP. Unfortunately, a similar transformation using 2,2-difluoro-Fuc failed. Interestingly, FKP could readily convert 2,2-difluoro-Fuc-1-P (**14**) obtained by chemical synthesis into **6**, which indicates that the first enzymatic step is most sensitive to chemical modifications.

### Inhibition of Six Human FUTs by Fluorinated GDP-Fuc Analogues **5** and **6**

Next, attention was focused on determining the inhibitory activity of the GDP-mono- (**5**) and di- (**6**) fluoro-Fuc derivatives. The disaccharide LacNAc **8** was used as the acceptor for FUT1, 3, 5, 6, and 9, while the biantennary glycan **7** was employed as the acceptor for FUT8.^8,12,33−40^ Kinetic parameters were obtained by keeping the acceptors (**7** and **8**) at saturating concentrations while varying the concentrations of GDP-Fuc (3 to 100 μM) and fluorinated inhibitors **5** and **6** (3 to 300 μM). Following an incubation period of 1 h, reaction conversions were determined by using a commercially available GDP-Glo glycosyltransferase assay kit. The amount of enzyme used was determined by test reactions to ensure that the reaction remained within the linear rate range. The data was analyzed using Prism and fitted by nonlinear regression. The results are summarized in Table 1.

**Table 1:** Inhibition of Human FUTs by GDP-2-F-Fuc and GDP-2,2-di-F-Fuc

	FUT1	FUT3	FUT5	FUT6	FUT8	FUT9
GDP-Fuc (*K*_m_, μM)	19.7 ± 1.9	3.1 ± 0.6	10.2 ± 0.8	7.9 ± 0.7	33.8 ± 2.5	8.0 ± 0.76
GDP-F-Fuc (**5**) (*K_i_*, μM)	35.9 ± 10.4	17.9 ± 1.9	43.4 ± 4.3	26.3 ± 2.5	111.8 ± 8.5	27.8 ± 1.7
GDP-di-F-Fuc (**6**) (*K_i_*, μM)	20.5 ± 2.2	11.4 ± 1.9	26.0 ± 2.1	25.0 ± 2.7	116.0 ± 17.5	34.2 ± 2.6

The *K*_m_ values for the various FUTs differ considerably; FUT3 has the smallest value and FUT8 the largest, while the others have intermediate inhibitory activities. Furthermore, the results demonstrate that GDP-2,2-di-F-Fuc (**6**) is a competitive inhibitor for all examined FUTs. Interestingly, **5** and **6** had similar inhibitory activities for FUT1, 3, 5, 6, and 8 whereas the *K_i_* value for FUT8 is substantially higher.

**Figure 2:**
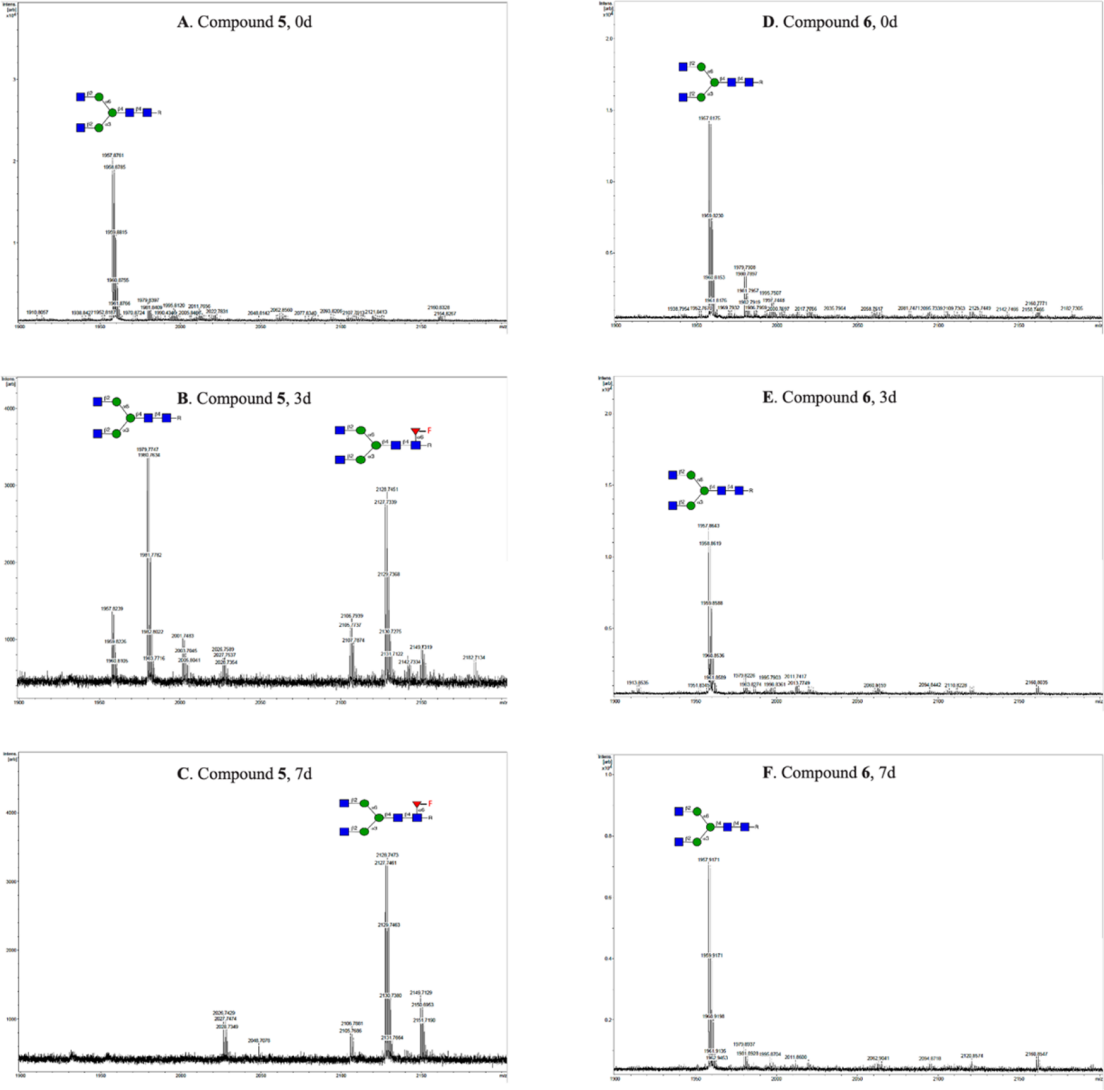
MALDI-TOF analysis of two parallel FUT8-catalyzed transfer reactions in the presence of 100 mM Tris buffer and 10 mM MgCl_2_. (A–C), time points for compound **5** as donor substrate. (D–F), time points for compound **6** as donor.

A limitation of compound **5** is its slow activity as a substrate for certain FUTs, particularly FUT8.^14^ One of the aims for introducing the second fluorine atom at **6** is to increase the stability of the glycosidic bond, preventing transfer. To validate this hypothesis, we set up two parallel transfer reactions catalyzed by FUT8 using *N*-glycan **7** as an acceptor (300 μM) and compounds **5** and **6** (750 μM) as a donor. The possible transfer of fluorinated fucose was monitored using MALDI-TOF MS at 0, 3, and 7 days (Figure 2). After 3 days, a substantial amount of **5** had been transferred to 7, while, after 7 days, the reaction had gone to near completion. In contrast, no product formation was observed for compound **6** and even after 7 days, no observable signals emerged for the fucosylated product, indicating that GDP-di-F-Fuc does not act as a donor.

### Inhibition of Cell Surface Fucosylation

Although fluorinated GDP-Fuc analogues exhibit potent inhibitory activity, these compounds cannot be employed for cell-based inhibition studies due to poor cell permeability. A prodrug strategy based on the biosynthetic pathway of nucleotide sugars has been developed to improve the cell permeability of Fuc analogues.^14−18^ In this respect, GDP-Fuc can be biosynthesized by *de novo* and salvage pathway (Figure 2).^1,41−44^ Quantitative examination of the fucose metabolism in HeLa cells has shown that over 90% of GDP-Fuc originates from *de novo* biosynthesis.^45^ It starts by conversion of GDP-mannose into GDP-4-keto-6-deoxymannose catalyzed by GDP-mannose-4,6-dehydratase (GMD) which is converted into GDP-Fuc by a dual functional epimerase-reductase known as the FX protein.^44^ GDP-Fuc acts as an inhibitor of GMD thereby blocking the *de novo* pathway and regulating GDP-Fuc levels. In the salvage pathway, GDP-Fuc is biosynthesized from free fucose derived from extracellular or lysosomal sources by anomeric phosphorylation by fucose kinase to give Fuc-1-phosphate that is converted to GDP-Fuc by GDP-Fuc pyrophosphorylase (GFPP).

**Figure 3:**
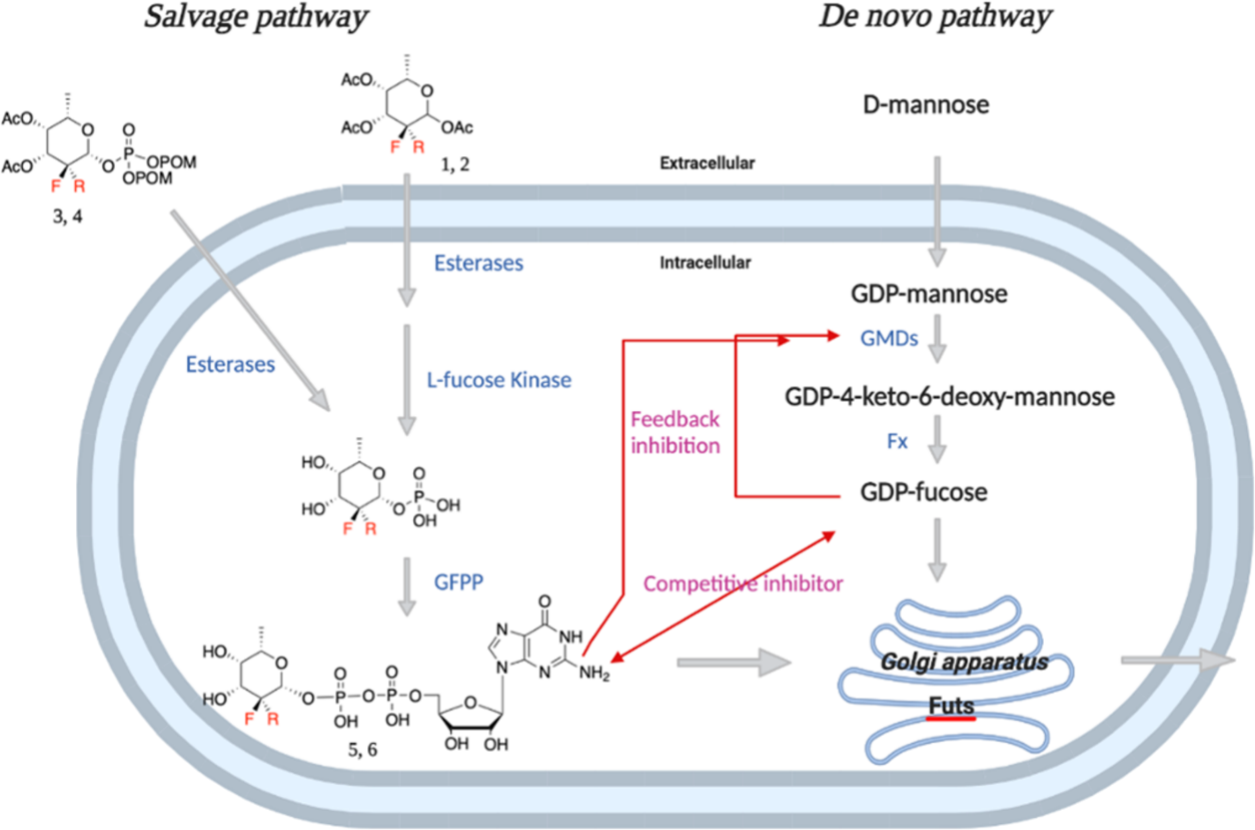
Metabolic pathway of fluorinated inhibitors in cells.

The salvage pathway has been exploited to convert fucose analogues that can penetrate the cell membrane into the corresponding GDP-Fuc derivatives.^14−18^ Here, we tested compounds **1** and **2** as inhibitors of fucosylation of cell surface glycoconjugates. After cell uptake, esterases can remove the acetyl esters, and the resulting compounds can then be converted into the corresponding GDP-Fuc derivatives by fucose kinase and GFPP (Figure 3). Fucose kinase has restricted substrate specificity and therefore protected monophosphate fucose derivatives have been developed that can cross the cell membrane^14,16,25^ Therefore, we also examined POM carbonates of 2-F-Fuc-1-P (**3**) and 2-di-F-Fuc-1-P (**4**) as precursors of the corresponding monophosphates.

**Figure 4:**
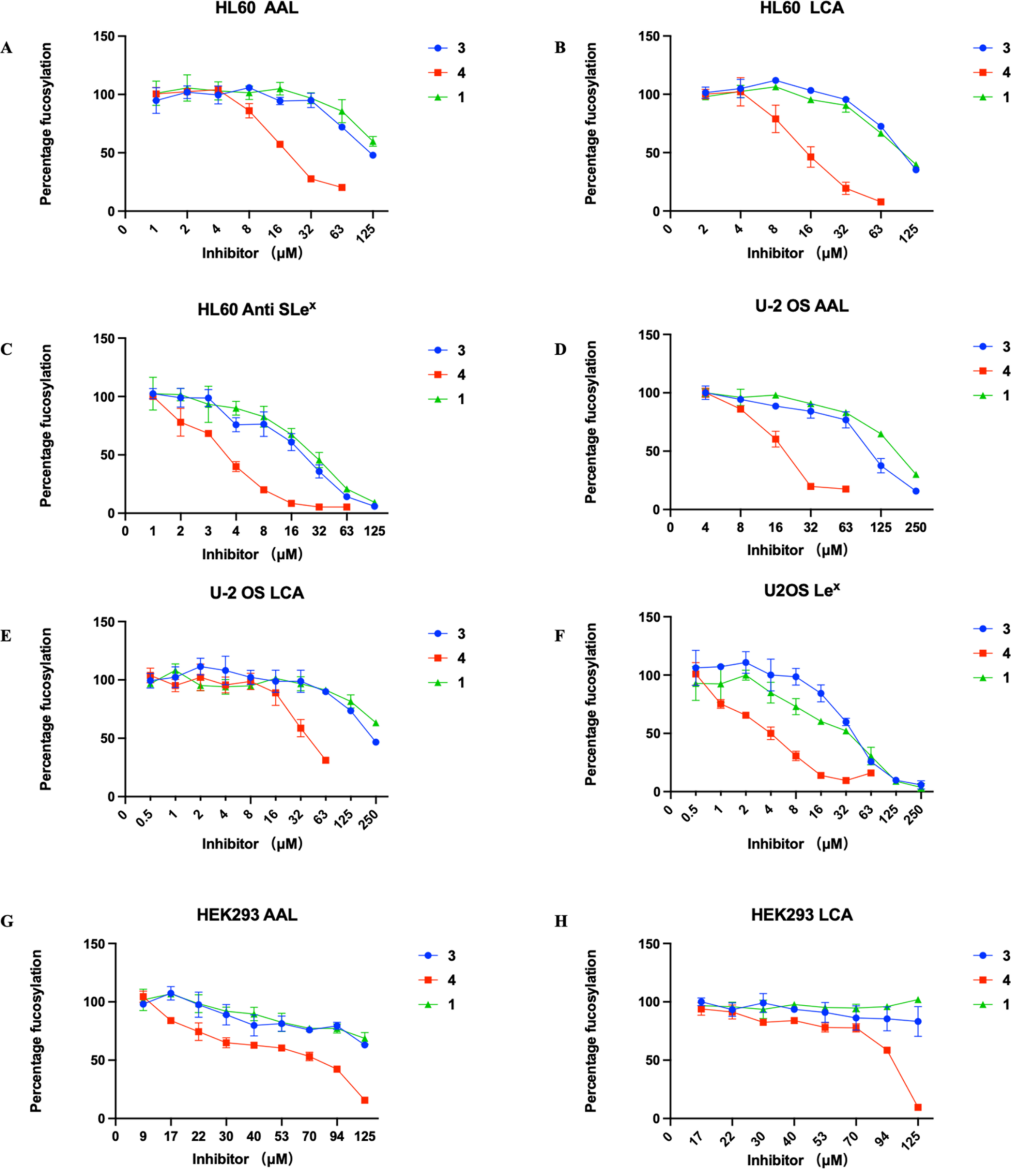
Fluorinated Fuc prodrugs act as fucosyl transferase inhibitors of cells. Cells were subjected to prodrugs at concentrations ranging from 0.5 to 250 μM or in DMSO control for 3 days. The data were normalized to cells treated with DMSO only as 100% fucosylation and unstained cells as 0%. The data presented are indicative of three independent experiments conducted in triplicate, illustrating the mean ± s.d. values. Error bars are displayed for each data set.

The inhibitory activity of the compounds **1**–**4** was evaluated in HL60, U-2 OS, and HEK293 cell lines.^46^ Cells were treated with prodrugs at concentrations ranging from 0.5 to 250 μM or in a DMSO control. After 3 days, the cells were harvested and analyzed using flow cytometry to evaluate the expression of cell surface fucosides by employing *Aleuria aurantia* lectin (AAL) and *Lens culinaris* agglutinin (LCA) and anti-Le*^x^* and SLe*^x^* antibodies (Figure 4).^14,19,47−49^ AAL has broad binding properties and preferentially binds to α1,3-, α1,2-, α1,4-, and α1,6-linked fucosides of *N*-acetyl-lactosamine. Per-*O*-acetyl-2,2-di-F-Fuc (**2**) did not show any reduction in cell surface fucosylation, while 2,2-di-F-Fuc-1-P-POM (**4**) exhibited potent inhibition. This observation indicates that fucose kinase cannot phosphorylate 2,2-di-F-Fuc, whereas 2-F-Fuc is a proper substrate for this enzyme. At a concentration of 32 μM, compound **4** suppressed most all cell surface fucosylation, as demonstrated by a lack of binding of AAL and LCA in the two cell lines. In contrast, a comparable reduction in cell surface fucosylation by compounds **1** and **3** required concentrations higher than 125 μM. It is worth noting that for both HL60 and U-2 OS, a lower concentration of compound **4** was required to inhibit Le*^x^*/SLe*^x^* expression (8 μM) compared to core fucosylation which was detected by LCA. This observation agrees with the relatively high *K*_m_ value for FUT8 which is responsible for core fucosylation. We also investigated the effects of the compounds on cell proliferation and viability,^50,51^ and no effects were observed at concentrations lower than 125 μM (Figure S4).

### Glycan Profiling of Cells Treated with Inhibitor

**Figure 5:**
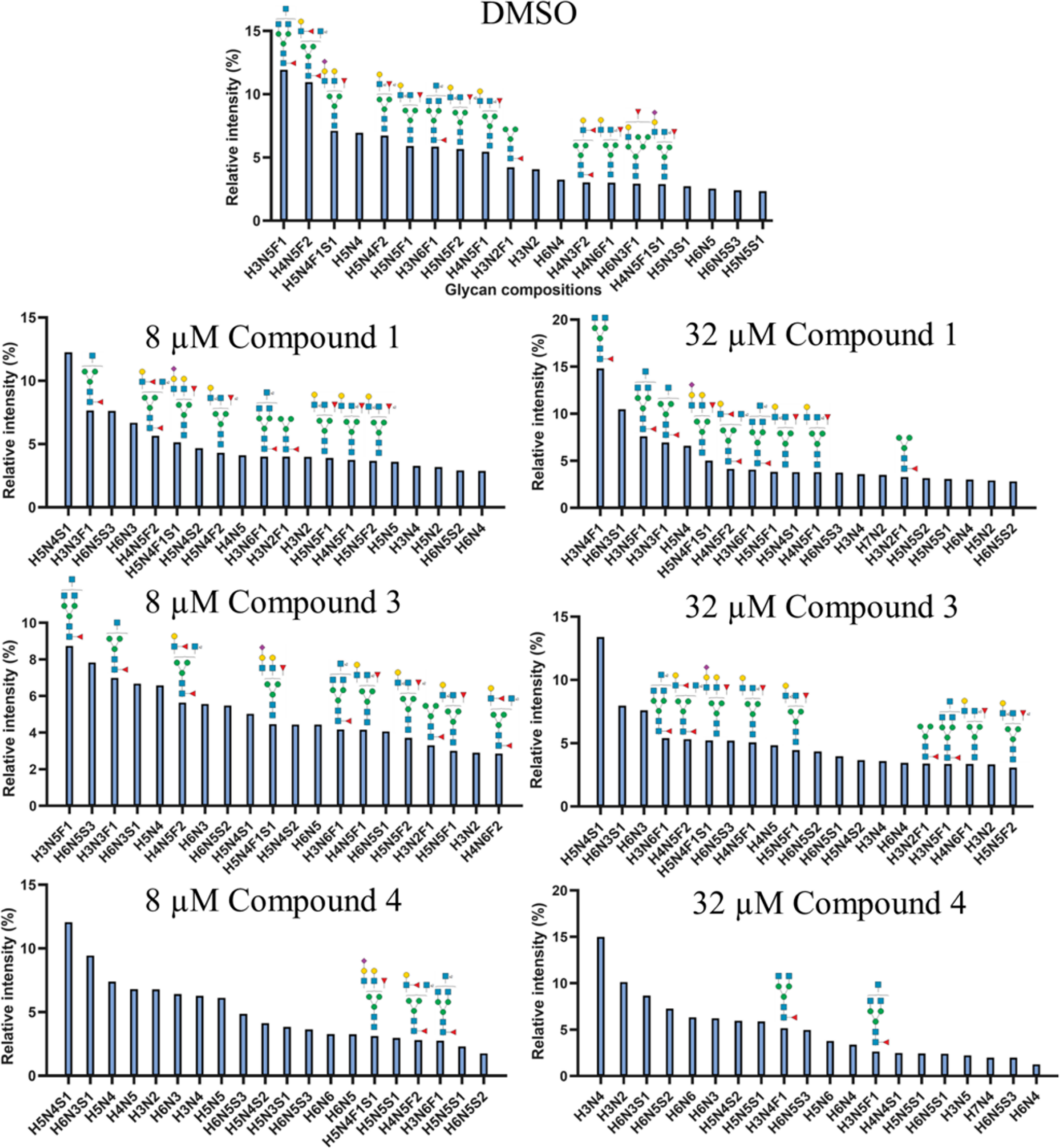
*N*-Glycosylation profiles of HL-60 cells treated with compounds **1**, **3**, and **4**. The proposed putative fucosylated *N*-glycan structures are based on a monosaccharide composition. Only fucosylated structures are shown to highlight the reduction in fucosylation. The compositions of the other glycans are presented as letter codes (H: hexose; N: *N*-acetylhexosamine; F: deoxyhexose; S: *N*-acetylneuraminic acid). The full list of glycan compositions is provided in the SI.

To further investigate the impact of the fluorinated fucose derivatives on cell surface fucosylation, we performed LC-MS analysis of *N*-glycans released from HL-60 cells.^52^ Thus, the cells were treated with 8 and 32 μM compounds **1**, **3**, and **4** and DMSO control. After 3 days, the cells were harvested and subjected to PNGase F treatment to liberate the *N*-glycans, followed by labeling with procainamide for positive-mode detection. The top 20 most abundant *N*-glycans for each sample are shown in Figure 5 to illustrate the change in the *N*-glycan profile (see the Supporting Information for a full list of glycan compositions). Only for the fucosylated glycans, putative compositional structures are depicted to highlight changes in fucosylation.

At low concentrations of inhibitors, compounds **1** and **3** had minimal influence on the HL-60 cell *N*-glycan profile. In contrast, compound **4** displayed a notable relative decrease in fucosylated structures with only low levels of core fucosylation detected at the highest concentration of 32 μM. It is also important to point out that there are differences in the structures of the 20 most abundant glycans across different conditions, which is possibly due to competition in enzymatic glycan modifications and, for example, core fucosylation can affect further glycan biosynthesis.^53^

In addition to the compositional analysis, a comparison of in-source fragment ions of fucosylated terminal *N*-glycan epitopes showed a drastic decrease in terminal fucosyl-LacNAc (i.e., Le*^x^*, H-antigen) and SLe*^x^*. This difference was most evident in compound **4**-treated cells (see Figure S5). Noteworthy is that the abundance of sialyl-LacNAc in-source fragments showed a slight increase compared to the DMSO control.

### Nucleotide Sugar Analysis of Cells Treated with **1**, **3**, and **4**

**Figure 6:**
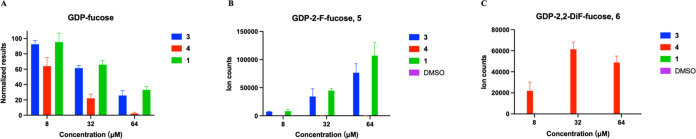
Sugar nucleotide quantification of HL60 cells treated with compounds **1**, **3**, and **4**. HL60 cells were treated with **1**, **3**, or **4** and DMSO control for 3 days. (A), Relative concentrations of GDP-Fuc in cell lysates. The data was normalized to cells incubated with DMSO at 100%. (B, C) Ion counts of GDP-2-F-Fuc and GDP-2,2-di-F-Fuc detected by LC-MS in lysates of cells treated with **1**, **3**, **4**, and DMSO as control. The data presented are of three independent experiments conducted in triplicate, illustrating mean ± s.d. values. Error bars are displayed for each data set. The LC-MS traces are provided in the SI.

The observation that GDP-2-F-Fuc (**5**) and GDP-2,2-di-F-Fuc (**6**) exhibit small differences in *K_i_* values for the various human fucosyl transferases, while the corresponding phosphate prodrugs **3** and **4** showed relatively large differences in inhibition of cell surface fucosylation, indicates that they have different feedback inhibitory activities. To validate this hypothesis, quantitative sugar nucleotide analysis was performed by LC-MS approach according to a previously reported methodology.^54^ Different concentrations of **1**, **3**, and **4** were administered to HL60 cells in culture medium, and after 3 days, cell lysates were collected for LC-MS analysis. GDP-Fuc, GDP-2-F-Fuc (**5**), and GDP-2,2-di-F-Fuc (**6**) were used as internal standards to quantify the relative levels of the corresponding sugar nucleotides in the cells (Figure 6). As anticipated, the intracellular concentration of GDP-Fuc decreases with increasing concentrations of inhibitors **1**, **3**, and **4** (Figure 6A). The most substantial reduction was observed with **4**, and at 8 μM, it already induced a noticeable impact on the GDP-Fuc level, while at 32 μM, it reduced the GDP-Fuc concentration to 20% compared to control, and at 64 μM, the presence of GDP-Fuc was almost undetectable. GDP-2-F-Fuc (**5**) was detected in cell samples treated with **1** and **3**, and GDP-2,2-di-F-Fuc (**6**) was detected in cell samples exposed to **4** (Figure 6B,C). Across the concentration range of 8–64 μM, the concentration of **5** increased with increasing concentrations of **1** and **3**. The data also indicates that **4** is more readily converted into GDP-Fuc analogue **6** compared to the conversion of **1** and **3** into GDP-Fuc derivative **5** and exhibits more potent feedback inhibition of GDP-Fuc biosynthesis.

## Conclusions

A synthetic methodology has been developed for the preparation of GDP-2,2-di-F-Fuc (**6**) and a corresponding prodrug (**4**), and the resulting compounds have been examined for the inhibition of fucosylation of cell surface glycoconjugates. The synthesis of the compounds was challenging due to the presence of two fluorine atoms at C-2 of fucose. The most efficient approach entailed the preparation of a lactol of 2,2-di-Fuc that was employed as a nucleophile in the presence of a weak base to react with tetrabenzyl pyrophosphate to provide the corresponding anomeric phosphate. After removal of the protecting groups, the resulting anomeric monophosphate could be converted into GDP-2,2-di-F-Fuc (**6**) by condensation with GMP-morpholidate. In addition, a phosphate prodrug (**4**) was prepared by the conversion of the monophosphate into a bis(pivaloyloxymethyl) (POM) ester. Similar compounds were prepared having only one F atom of C-2 fucose (**3** and **5**). It was found that GDP-2-F-Fuc (**5**) and GDP-2,2-di-F-Fuc (**6**) have similar inhibitory activities for various human fucosyl transferases. While slow transfer of GDP-2-F-Fuc (**5**) was observed, the corresponding GDP-2,2-di-F-Fuc (**6**) could not modify glycosyl acceptors, even after a prolonged period of incubation. This is due to the strong electronegativity of the two fluorine atoms that stabilizes the anomeric phosphate.^20^ Interestingly, the monofluorinated (**3**) and difluorinated (**4**) phosphate prodrugs showed substantial differences in potency of inhibiting cell surface fucosylation, and difluorinated derivative **4** is more active at lower concentrations. Examination of the concentrations of GDP-Fuc by LC-MS indicated that **4** has greater feedback inhibitory activity compared to the monofluorinated analogue. These studies also confirmed that intracellularly, **4** is converted into GDP-2,2-di-F-Fuc (**6**). Another surprising observation was that lower concentrations of **4** are required to block the biosynthesis of Le*^x^* and SLe*^x^* compared to the core fucosylation of *N*-glycans. This is attributed to differences in *K*_m_ values of the various FUTs and in particular FUT8, which is responsible for core fucosylation has the highest *K*_m_ value of the examined fucosyl transferases and thus is most sensitive to lower levels of GDP-Fuc caused by the application of the inhibitors. Thus, it is likely that inhibition of cell surface fucosylation by compound **4** is due to intracellular conversion into GDP-2,2-di-F-Fuc (**6**), which can inhibit various fucosyl transferases but also prevents the *de novo* biosynthesis of GDP-Fuc.

Previously, a series of fluorinated fucose analogues was reported that was examined for their effect on cell proliferation of human colon cancer and angiogenetic cells.^25^ It was found that the earlier reported 2-F-Fuc and 6-F-Fuc had only a marginal effect, whereas 6,6-di-F-Fuc and 6,6,6-tri-F-Fuc showed significant inhibition of cell proliferation. The GDP derivatives of 6,6-di-F-Fuc and 6,6,6-tri-F-Fuc were also prepared, and it was found that they have a lower inhibitory activity for FUT8 compared to GDP-2-F-Fuc. Thus, it was suggested that in addition to inhibiting FUT8, 6-di-F-Fuc, and 6,6,6-tri-F-Fuc achieve inhibition of cell proliferation by targeting other steps of the fucosylation biosynthetic pathway. 2,2-Di-F-Fuc was also investigated and was found to poorly inhibit cell proliferation. It was attempted to enzymatically convert this compound into the corresponding GDP derivative by using FKP, which failed. Based on our observations, 2,2-di-F-Fuc is a poor substrate for anomeric phosphorylation by FKP, which explains the previously observed low cellular activity. This lack of activity can be overcome by employing a phosphate prodrug approach such as in compound **4**. The attraction of prodrug **4** is that the corresponding GDP derivative is an equally potent inhibitor for various fucosyl transferases compared to 2-F-Fuc, but appears to be more active in inhibiting the *de novo* biosynthesis of GDP-Fuc. Collectively, the data indicates that the structure–activity relationship differs for feedback and fucosyl transfer inhibition. Recently, it was shown that β-carbafucose can also be employed by the fucose salvage pathway to form GDP-carbafucose.^13^ This compound cannot form an oxocarbenium ion-like transition state, thereby acting as an inhibitor for fucosyl transferases.^12^ Treatment of CHO cells with β-carbafucose leads to a dose-dependent reduction in core fucosylation of the therapeutic antibody Herceptin without affecting cell growth and transfer of the modified fucose derivative, highlighting the potential therapeutic use of fucose analogues.

## Supporting Information Available

Synthetic protocols; compound characterization; cell experimental; and NMR spectra (PDF)

## References

[ref1] Becker D. J.; Lowe J. B. Fucose: biosynthesis and biological function in mammals. Glycobiology 2003, 13, 41R–53R. 10.1093/glycob/cwg054. 12651883

[ref2] Miyoshi E.; Moriwaki K.; Terao N.; Tan C. C.; Terao M.; Nakagawa T.; Matsumoto H.; Shinzaki S.; Kamada Y. Fucosylation is a promising target for cancer diagnosis and therapy. Biomolecules 2012, 2, 34–45. 10.3390/biom2010034. 24970126 PMC4030867

[ref3] Li J.; Hsu H.-C.; Mountz J. D.; Allen J. G. Unmasking fucosylation: From cell adhesion to immune system regulation and diseases. Cell Chem. Biol. 2018, 25, 499–512. 10.1016/j.chembiol.2018.02.005. 29526711

[ref4] Ma B.; Simala-Grant J. L.; Taylor D. E. Fucosylation in prokaryotes and eukaryotes. Glycobiology 2006, 16, 158R–184R. 10.1093/glycob/cwl040. 16973733

[ref5] Murray B. W.; Takayama S.; Schultz J.; Wong C. H. Mechanism and specificity of human alpha-1,3-fucosyltransferase V. Biochemistry 1996, 35, 11183–11195. 10.1021/bi961065a. 8780523

[ref6] Burkart M. D.; Vincent S. P.; Duffels A.; Murray B. W.; Ley S. V.; Wong C. H. Chemo-enzymatic synthesis of fluorinated sugar nucleotide: useful mechanistic probes for glycosyltransferases. Bioorg. Med. Chem. 2000, 8, 1937–1946. 10.1016/S0968-0896(00)00139-5. 11003139

[ref7] Hosoguchi K.; Maeda T.; Furukawa J.; Shinohara Y.; Hinou H.; Sekiguchi M.; Togame H.; Takemoto H.; Kondo H.; Nishimura S. An efficient approach to the discovery of potent inhibitors against glycosyltransferases. J. Med. Chem. 2010, 53, 5607–5619. 10.1021/jm100612r. 20684602

[ref8] Lin Y. N.; Stein D.; Lin S. W.; Chang S. M.; Lin T. C.; Chuang Y. R.; Gervay-Hague J.; Narimatsu H.; Lin C. H. Chemoenzymatic synthesis of GDP-L-fucose derivatives as potent and selective α-1,3-fucosyltransferase inhibitors. Adv. Synth. Catal. 2012, 354, 1750–1758. 10.1002/adsc.201100940.

[ref9] Bryan M. C.; Lee L. V.; Wong C. H. High-throughput identification of fucosyltransferase inhibitors using carbohydrate microarrays. Bioorg. Med. Chem. Lett. 2004, 14, 3185–3188. 10.1016/j.bmcl.2004.04.001. 15149672

[ref10] Rillahan C. D.; Brown S. J.; Register A. C.; Rosen H.; Paulson J. C. High-throughput screening for inhibitors of sialyl- and fucosyltransferases. Angew. Chem., Int. Ed. 2011, 50, 12534–12537. 10.1002/anie.201105065. PMC3245354 22095645

[ref11] Niu X.; Fan X.; Sun J.; Ting P.; Narula S.; Lundell D. Inhibition of fucosyltransferase VII by gallic acid and its derivatives. Arch. Biochem. Biophys. 2004, 425, 51–57. 10.1016/j.abb.2004.02.039. 15081893

[ref12] Norris A. J.; Whitelegge J. P.; Strouse M. J.; Faull K. F.; Toyokuni T. Inhibition kinetics of carba- and C-fucosyl analogues of GDP-fucose against fucosyltransferase V:: implication for the reaction mechanism. Bioorg. Med. Chem. Lett. 2004, 14, 571–573. 10.1016/j.bmcl.2003.12.003. 14741245

[ref13] Gilormini P. A.; Thota V. N.; Fers-Lidou A.; Ashmus R. A.; Nodwell M.; Brockerman J.; Kuo C. W.; Wang Y.; Gray T. E.; Nitin; McDonagh A. W.; Guu S. Y.; Ertunc N.; Yeo D.; Zandberg W. F.; Khoo K. H.; Britton R.; Vocadlo D. J. A metabolic inhibitor blocks cellular fucosylation and enables production of afucosylated antibodies. Proc. Natl. Acad. Sci. U.S.A. 2024, 121, e2314026121 10.1073/pnas.2314026121. 38917011 PMC11228515

[ref14] Rillahan C. D.; Antonopoulos A.; Lefort C. T.; Sonon R.; Azadi P.; Ley K.; Dell A.; Haslam S. M.; Paulson J. C. Global metabolic inhibitors of sialyl- and fucosyltransferases remodel the glycome. Nat. Chem. Biol. 2012, 8, 661–668. 10.1038/nchembio.999. 22683610 PMC3427410

[ref15] Zandberg W. F.; Kumarasamy J.; Pinto B. M.; Vocadlo D. J. Metabolic inhibition of sialyl-Lewis X biosynthesis by 5-thiofucose remodels the cell surface and impairs selectin-mediated cell adhesion. J. Biol. Chem. 2012, 287, 40021–40030. 10.1074/jbc.M112.403568. 23019334 PMC3501042

[ref16] Allen J. G.; Mujacic M.; Frohn M. J.; Pickrell A. J.; Kodama P.; Bagal D.; San Miguel T.; Sickmier E. A.; Osgood S.; Swietlow A.; Li V.; Jordan J. B.; Kim K. W.; Rousseau A. C.; Kim Y. J.; Caille S.; Achmatowicz M.; Thiel O.; Fotsch C. H.; Reddy P.; McCarter J. D. Facile modulation of antibody fucosylation with small molecule fucostatin inhibitors and cocrystal structure with GDP-mannose 4,6-dehydratase. ACS Chem. Biol. 2016, 11, 2734–2743. 10.1021/acschembio.6b00460. 27434622

[ref17] Pijnenborg J. F. A.; Visser E. A.; Noga M.; Rossing E.; Veizaj R.; Lefeber D. J.; Bull C.; Boltje T. J. Cellular Fucosylation Inhibitors Based on Fluorinated Fucose-1-phosphates*. Chem. - Eur. J. 2021, 27, 4022–4027. 10.1002/chem.202005359. 33336886 PMC7986151

[ref18] Pijnenborg J. F. A.; Rossing E.; Merx J.; Noga M. J.; Titulaer W. H. C.; Eerden N.; Veizaj R.; White P. B.; Lefeber D. J.; Boltje T. J. Fluorinated rhamnosides inhibit cellular fucosylation. Nat. Commun. 2021, 12, 7024 10.1038/s41467-021-27355-9. 34857733 PMC8640046

[ref19] Okeley N. M.; Alley S. C.; Anderson M. E.; Boursalian T. E.; Burke P. J.; Emmerton K. M.; Jeffrey S. C.; Klussman K.; Law C. L.; Sussman D.; Toki B. E.; Westendorf L.; Zeng W.; Zhang X.; Benjamin D. R.; Senter P. D. Development of orally active inhibitors of protein and cellular fucosylation. Proc. Natl. Acad. Sci. U.S.A. 2013, 110, 5404–5409. 10.1073/pnas.1222263110. 23493549 PMC3619284

[ref20] Braun C.; Brayer G. D.; Withers S. G. Mechanism-based inhibition of yeast alpha-glucosidase and human pancreatic alpha-amylase by a new class of inhibitors. 2-Deoxy-2,2-difluoro-alpha-glycosides. J. Biol. Chem. 1995, 270, 26778–26781. 10.1074/jbc.270.45.26778. 7592915

[ref21] Pradere U.; Garnier-Amblard E. C.; Coats S. J.; Amblard F.; Schinazi R. F. Synthesis of nucleoside phosphate and phosphonate prodrugs. Chem. Rev. 2014, 114, 9154–9218. 10.1021/cr5002035. 25144792 PMC4173794

[ref22] Burkart M. D.; Zhang Z. Y.; Hung S. C.; Wong C. H. A new method for the synthesis of fluoro-carbohydrates and glycosides using selectfluor. J. Am. Chem. Soc. 1997, 119, 11743–11746. 10.1021/ja9723904.

[ref23] Francisco C. G.; Gonzalez C. C.; Kennedy A. R.; Paz N. R.; Suarez E. Fragmentation of carbohydrate anomeric alkoxyl radicals: new synthesis of chiral 1-fluoro-1-halo-1-iodoalditols. Chem. - Eur. J. 2008, 14, 6704–6712. 10.1002/chem.200800734. 18576400

[ref24] Bucher C.; Gilmour R. Fluorine-directed glycosylation. Angew. Chem., Int. Ed. 2010, 49, 8724–8728. 10.1002/anie.201004467. 20886497

[ref25] Dai Y.; Hartke R.; Li C.; Yang Q.; Liu J. O.; Wang L. X. Synthetic fluorinated I-fucose analogs inhibit proliferation of cancer cells and primary endothelial cells. ACS Chem. Biol. 2020, 15, 2662–2672. 10.1021/acschembio.0c00228. 32930566 PMC10901565

[ref26] Xu Y.; Prestwich G. D. Concise synthesis of acyl migration-blocked 1,1-difluorinated analogues of lysophosphatidic acid. J. Org. Chem. 2002, 67, 7158–7161. 10.1021/jo0203037. 12354017

[ref27] Zhu J. S.; McCormick N. E.; Timmons S. C.; Jakeman D. L. Synthesis of alpha-deoxymono and difluorohexopyranosyl 1-phosphates and kinetic evaluation with thymidylyl- and guanidylyltransferases. J. Org. Chem. 2016, 81, 8816–8825. 10.1021/acs.joc.6b01485. 27576508

[ref28] Piscelli B. A.; Sanders W.; Yu C.; Al Maharik N.; Lebl T.; Cormanich R. A.; O’Hagan D. Fluorine-induced *pseudo*-anomeric effects in methoxycyclohexanes through electrostatic 1,3-diaxial interactions. Chem. - Eur. J. 2020, 26, 11989–11994. 10.1002/chem.202003058. 32588927 PMC7540582

[ref29] Cumpstey I. On a so-called ″kinetic anomeric effect″ in chemical glycosylation. Org. Biomol. Chem. 2012, 10, 2503–2508. 10.1039/c2ob06696c. 22336963

[ref30] Nguyen H.; Zhu D.; Li X.; Zhu J. Stereoselective construction of beta-mannopyranosides by anomeric o-alkylation: Synthesis of the trisaccharide core of *N*-linked glycans. Angew. Chem., Int. Ed. 2016, 55, 4767–4771. 10.1002/anie.201600488. 26948686

[ref31] Yao H.; Vu M. D.; Liu X. W. Recent advances in reagent-controlled stereoselective/stereospecific glycosylation. Carbohydr. Res. 2019, 473, 72–81. 10.1016/j.carres.2018.10.006. 30641292

[ref32] Wang W.; Hu T.; Frantom P. A.; Zheng T.; Gerwe B.; Del Amo D. S.; Garret S.; Seidel R. D. III; Wu P. Chemoenzymatic synthesis of GDP-L-fucose and the Lewis X glycan derivatives. Proc. Natl. Acad. Sci. U.S.A. 2009, 106, 16096–16101. 10.1073/pnas.0908248106. 19805264 PMC2752511

[ref33] Palcic M. M.; Heerze L. D.; Srivastava O. P.; Hindsgaul O. A bisubstrate analog inhibitor for alpha(1->2)-fucosyltransferase. J. Biol. Chem. 1989, 264, 17174–17181. 10.1016/S0021-9258(18)71475-0. 2551897

[ref34] Murray B. W.; Wittmann V.; Burkart M. D.; Hung S. C.; Wong C. H. Mechanism of human alpha-1,3-fucosyltransferase V: glycosidic cleavage occurs prior to nucleophilic attack. Biochemistry 1997, 36, 823–831. 10.1021/bi962284z. 9020780

[ref35] Lee L. V.; Mitchell M. L.; Huang S. J.; Fokin V. V.; Sharpless K. B.; Wong C. H. A potent and highly selective inhibitor of human alpha-1,3-fucosyltransferase via click chemistry. J. Am. Chem. Soc. 2003, 125, 9588–9589. 10.1021/ja0302836. 12904015

[ref36] Galan M. C.; Venot A. P.; Phillips R. S.; Boons G. J. The design and synthesis of a selective inhibitor of fucosyltransferase VI. Org. Biomol. Chem. 2004, 2, 1376–1380. 10.1039/b317067e. 15105929

[ref37] Kotzler M. P.; Blank S.; Bantleon F. I.; Wienke M.; Spillner E.; Meyer B. Donor assists acceptor binding and catalysis of human alpha1,6-fucosyltransferase. ACS Chem. Biol. 2013, 8, 1830–1840. 10.1021/cb400140u. 23730796

[ref38] Seelhorst K.; Stacke C.; Ziegelmuller P.; Hahn U. *N*-glycosylations of human alpha1,3-fucosyltransferase IX are required for full enzyme activity. Glycobiology 2013, 23, 559–567. 10.1093/glycob/cws219. 23263199

[ref39] Seelhorst K.; Piernitzki T.; Lunau N.; Meier C.; Hahn U. Synthesis and analysis of potential alpha1,3-fucosyltransferase inhibitors. Bioorg. Med. Chem. 2014, 22, 6430–6437. 10.1016/j.bmc.2014.09.038. 25438767

[ref40] Tseng T. H.; Lin T. W.; Chen C. Y.; Chen C. H.; Lin J. L.; Hsu T. L.; Wong C. H. Substrate preference and interplay of fucosyltransferase 8 and N-acetylglucosaminyltransferases. J. Am. Chem. Soc. 2017, 139, 9431–9434. 10.1021/jacs.7b03729. 28678517

[ref41] Kaufman R. L.; Ginsburg V. The metabolism of L-fucose by HeLa cells. Exp. Cell Res. 1968, 50, 127–132. 10.1016/0014-4827(68)90400-X. 5650855

[ref42] Hinderlich S.; Berger M.; Blume A.; Chen H.; Ghaderi D.; Bauer C. Identification of human L-fucose kinase amino acid sequence. Biochem. Biophys. Res. Commun. 2002, 294, 650–654. 10.1016/S0006-291X(02)00541-7. 12056818

[ref43] Nakayama K.-i.; Maeda Y.; Jigami Y. Interaction of GDP-4-keto-6-deoxymannose-3,5-epimerase-4-reductase with GDP-mannose-4,6-dehydratase stabilizes the enzyme activity for formation of GDP-fucose from GDP-mannose. Glycobiology 2003, 13, 673–680. 10.1093/glycob/cwg099. 12881408

[ref44] Noda K.; Miyoshi E.; Gu J. G.; Gao C. X.; Nakahara S.; Kitada T.; Honke K.; Suzuki K.; Yoshihara H.; Yoshikawa K.; Kawano K.; Tonetti M.; Kasahara A.; Hori M.; Hayashi N.; Taniguchi N. Relationship between elevated FX expression and increased production of GDP-L-fucose, a common donor substrate for fucosylation in human hepatocellular carcinoma and hepatoma cell lines. Cancer Res. 2003, 63, 6282–6289. 14559815

[ref45] Yurchenco P. D.; Atkinson P. H. Equilibration of fucosyl glycoprotein pools in HeLa cells. Biochemistry 1977, 16, 944–953. 10.1021/bi00624a021. 843523

[ref46] Zhou Y.; Fukuda T.; Hang Q. L.; Hou S. C.; Isaji T.; Kameyama A.; Gu J. G. Inhibition of fucosylation by 2-fluorofucose suppresses human liver cancer HepG2 cell proliferation and migration as well as tumor formation. Sci. Rep. 2017, 7, 11563 10.1038/s41598-017-11911-9. 28912543 PMC5599613

[ref47] Böhm E.; Seyfried B. K.; Dockal M.; Graninger M.; Hasslacher M.; Neurath M.; Konetschny C.; Matthiessen P.; Mitterer A.; Scheiflinger F. Differences in *N*-glycosylation of recombinant human coagulation factor VII derived from BHK, CHO, and HEK293 cells. BMC Biotechnol. 2015, 15, 87 10.1186/s12896-015-0205-1. 26382581 PMC4574471

[ref48] Villalobos J. A.; Yi B. R.; Wallace I. S. 2-Fluoro-L-fucose is a metabolically incorporated inhibitor of plant cell wall polysaccharide fucosylation. PLoS One 2015, 10, e0139091 10.1371/journal.pone.0139091. 26414071 PMC4587364

[ref49] Kizuka Y.; Funayama S.; Shogomori H.; Nakano M.; Nakajima K.; Oka R.; Kitazume S.; Yamaguchi Y.; Sano M.; Korekane H.; Hsu T. L.; Lee H. Y.; Wong C. H.; Taniguchi N. High-sensitivity and low-toxicity fucose probe for glycan imaging and biomarker discovery. Cell Chem. Biol. 2016, 23, 782–792. 10.1016/j.chembiol.2016.06.010. 27447047

[ref50] Rixe O.; Fojo T. Is cell death a critical end point for anticancer therapies or is cytostasis sufficient?. Clin. Cancer Res. 2007, 13, 7280–7287. 10.1158/1078-0432.CCR-07-2141. 18094408

[ref51] Anttila J. V.; Shubin M.; Cairns J.; Borse F.; Guo Q.; Mononen T.; Vazquez-Garcia I.; Pulkkinen O.; Mustonen V. Contrasting the impact of cytotoxic and cytostatic drug therapies on tumour progression. PLoS Comput. Biol. 2019, 15, e1007493 10.1371/journal.pcbi.1007493. 31738747 PMC6886869

[ref52] Jang-Lee J.; North S. J.; Sutton-Smith M.; Goldberg D.; Panico M.; Morris H.; Haslam S.; Dell A. Glycomic profiling of cells and tissues by mass spectrometry: fingerprinting and sequencing methodologies. Methods Enzymol. 2006, 415, 59–86. 10.1016/S0076-6879(06)15005-3. 17116468

[ref53] Moremen K. W.; Tiemeyer M.; Nairn A. V. Vertebrate protein glycosylation: diversity, synthesis and function. Nat. Rev. Mol. Cell Biol. 2012, 13, 448–462. 10.1038/nrm3383. 22722607 PMC3934011

[ref54] van Scherpenzeel M.; Conte F.; Bull C.; Ashikov A.; Hermans E.; Willems A.; van Tol W.; Kragt E.; Noga M.; Moret E. E.; Heise T.; Langereis J. D.; Rossing E.; Zimmermann M.; Rubio-Gozalbo M. E.; de Jonge M. I.; Adema G. J.; Zamboni N.; Boltje T.; Lefeber D. J. Dynamic tracing of sugar metabolism reveals the mechanisms of action of synthetic sugar analogs. Glycobiology 2022, 32, 239–250. 10.1093/glycob/cwab106. 34939087 PMC8966471

